# Supplementary material for: Excited-State Energy Surfaces in Molecules Revealed by Impulsive Stimulated Raman Excitation Profiles
Source: J Phys Chem Lett. 2021 Sep 17;12(38):9239–47. doi: 10.1021/acs.jpclett.1c02209 (PMC8488957; doi:10.1021/acs.jpclett.1c02209)
Supplement: Supplementary file 1 — jz1c02209_si_001.pdf [file jz1c02209_si_001.pdf]

# Excited-state energy surfaces in molecules revealed by impulsive stimulated Raman excitation profiles: Supporting Information

Giovanni Batignani,[*,†,‡] Carlotta Sansone,[†] Carino Ferrante,[†,‡] Giuseppe Fumero,[†] Shaul Mukamel,[¶] and Tullio Scopigno[*,†,‡]

†*Dipartimento di Fisica, Universitá di Roma "La Sapienza", Roma, I-00185, Italy*

‡ *Istituto Italiano di Tecnologia, Center for Life Nano Science @Sapienza, Roma, I-00161, Italy*

¶*Department of Chemistry, University of California, Irvine, 92623, California, USA*

E-mail: giovanni.batignani@uniroma1.it; tullio.scopigno@uniroma1.it

# Impulsive Stimulated Raman Spectroscopy maps

In Figure S1 we report cresyl violet ISRS spectra obtained with a negatively chirped ($C_2$ = -80 fs$^2$) broadband resonant PP as a function of the time delay between Raman and probe pulses and the corresponding map in the frequency domain.

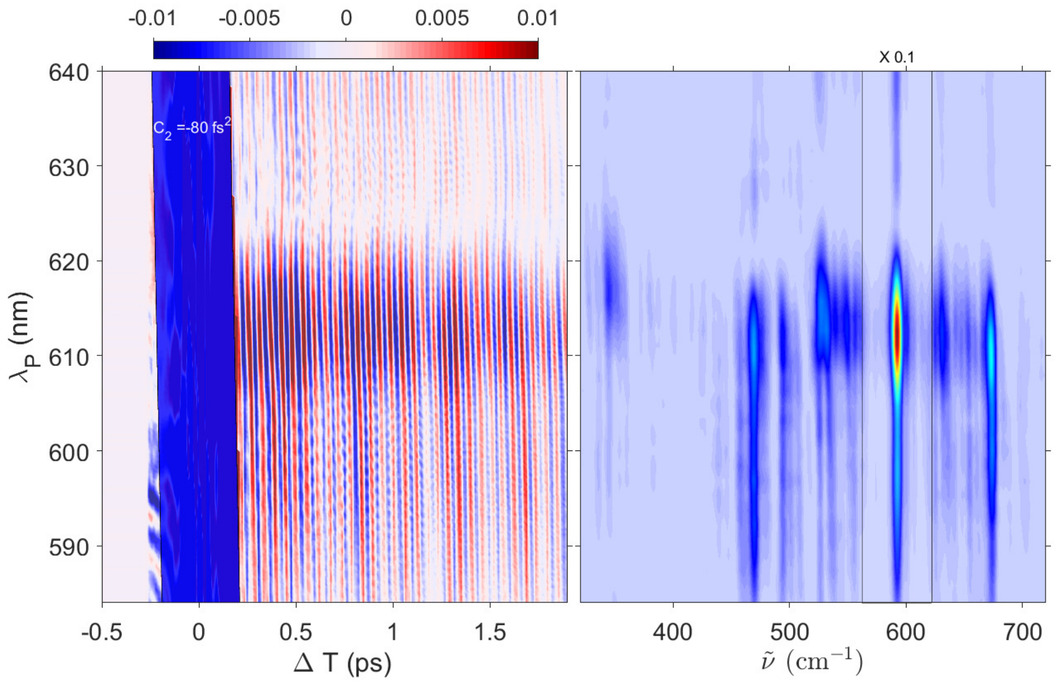


Figure S1: Cresyl Violet broadband two-color ISRS signal in time domain as a function of the probe wavelength $\lambda_P$ and RP-PP relative delay $\Delta T$ (left panel) measured for a negatively chirped PP ($C_2$ = -80 fs$^2$). The blue box indicates the region covered by the coherent artifact. The corresponding spectrum in the frequency domain has been obtained upon FFT and is reported in the right panel. The region around 592 cm$^{-1}$ has been scaled by a factor 0.1 to enhance the visibility of the weaker Raman modes.

# Chirp Measurement

The measurement of the PP chirp can be performed using the Coherent Artifact (CA) signal generated within the temporal overlap of two pulses, the Raman and the probe in our case,

inside the sample, identifying a wavelength dependent time delay between the two beams as

$$\tilde{\Delta T}_0(\lambda_P) = \frac{\int_{-\tau_{\lambda_s}}^{+\tau_{\lambda_s}} \Delta T |s_{CA}(\Delta T, \lambda_P)|^2 d\Delta T}{\int_{-\tau_{\lambda_s}}^{+\tau_{\lambda_s}} |s_{CA}(\Delta T, \lambda_P)|^2 d\Delta T} \tag{S1}$$

where $[-\tau_{\lambda_s}, +\tau_{\lambda_s}]$ are the wavelength-dependent time window containing the CA. An alternative approach to fit the wavelength dependent delay $\tilde{\Delta T}_0(\lambda_P)$ is to exploit the time-domain ISRS signal generated by the solvent. This latter can be isolated applying a filter centered around the 1040 cm$^{-1}$ Raman mode of the solvent to the frequency domain data and anti-Fourier transforming. The slope of the coherent oscillation in the resulting trace (reported in Fig. S2) can be exploited to extract $\tilde{\Delta T}_0(\lambda_P)$. Finally the probe chirp can be evaluated

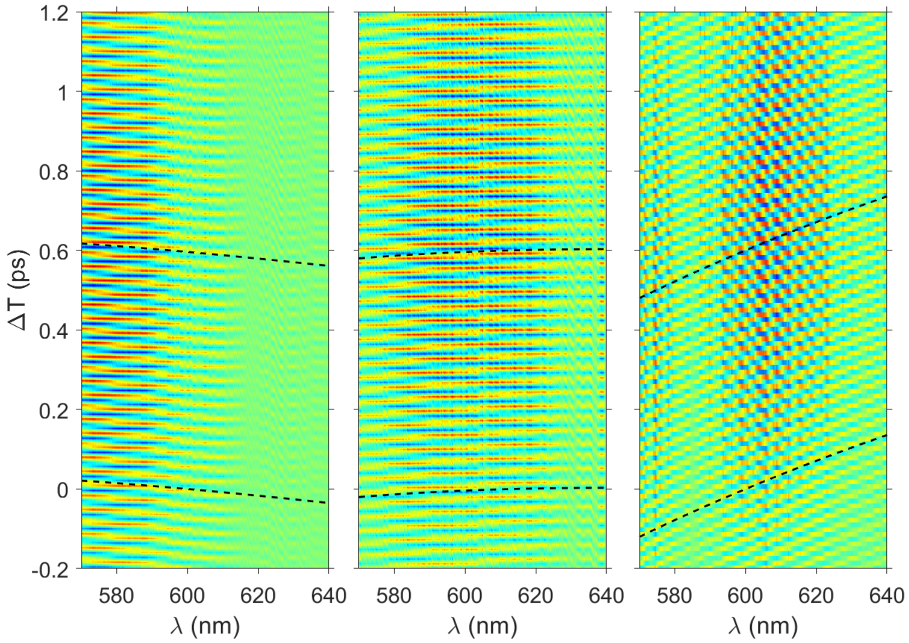


Figure S2: ISRS signal from methanol obtained upon applying a filter centered around the 1040 cm$^{-1}$ Raman mode and anti-Fourier transforming for three different values of the probe chirp ($C_2$ = -80, 40 and 360 fs$^2$, respectively). Dashed black lines show the PP chirp induced time delay $\tilde{\Delta T}_0(\lambda_P)$ between the Raman and probe pulses as a function of PP wavelength.

exploiting the relations:

$$\tilde{\Delta T}_0(\omega) = -\frac{d\phi}{d\omega},\ E_P(\omega) = E_P^{(0)}(\omega) e^{i\phi(\omega)},\ \phi(\omega) = \sum_n C_n(\omega - \omega_P)^n \tag{S2}$$

# Sample Absorption

In Fig. S3, the absorption spectrum of methanol and aqueous CV solutions are compared. Interestingly, the aqueous solution shows a blue shifted more pronounced shoulder, indicating higher displacements between the ground and the excited potential energy surfaces.

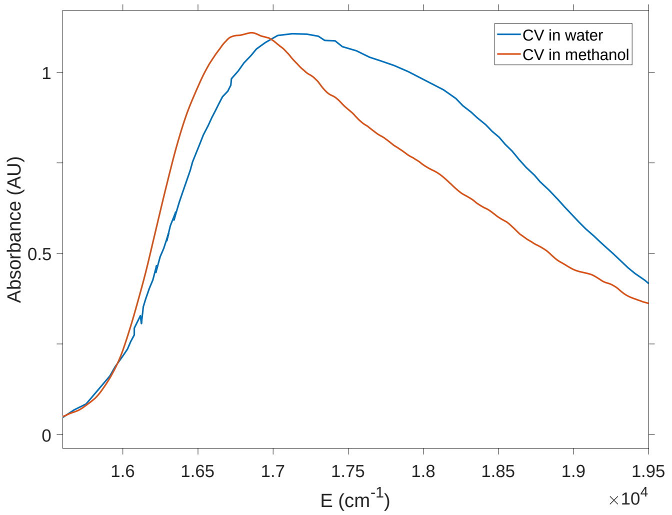


Figure S3: Absorption spectrum of Cresyl Violet dissolved in methanol is compared with the aqueous solution.[1]

# Derivation of the resonant ISRS signal

The third order polarization for a given vibrational mode $g'$ can be calculated in the time domain as[2–6]

$$P_A^{(3)}(t) = \left(\frac{i}{\hbar}\right)^3 \sum_{e_j e_k} \mu_{ge_j}\mu_{e_j g'}\mu_{g' e_k}\mu_{e_k g} \int_0^\infty d\tau_3 \int_0^\infty d\tau_2 \int_0^\infty d\tau_1$$
$$E_R(t-\tau_1-\tau_2-\tau_3)E_R^*(t-\tau_2-\tau_3)E_P(t-\tau_3)e^{-i\tilde{\omega}_{e_j g}\tau_1}e^{-i\tilde{\omega}_{g'g}\tau_2}e^{-i\tilde{\omega}_{e_k g}\tau_3} \quad \text{(S3)}$$

where $\mu_{ij}$ indicate the dipole moment between the $i$ and $j$ states and where we have considered only exponential dephasing of the $|i\rangle\langle j|$ coherences: $\tilde{\omega}_{ij} = \omega_i - \omega_j - i\gamma_{ij}$.

More generally, considering an additional inhomogeneous dephasing function $G(\tau)$ for the electronic coherence $|e_k\rangle\langle g|$, we have

$$P_A^{(3)}(t) = \left(\frac{i}{\hbar}\right)^3 \sum_{e_j e_k} \mu_{ge_j}\mu_{e_j g'}\mu_{g' e_k}\mu_{e_k g} \int_0^\infty d\tau_3 \int_0^\infty d\tau_2 \int_0^\infty d\tau_1$$
$$E_R(t-\tau_1-\tau_2-\tau_3)E_R^*(t-\tau_2-\tau_3)E_P(t-\tau_3)e^{-i\tilde{\omega}_{e_j g}\tau_1}G(\tau_1)e^{-i\tilde{\omega}_{g' g}\tau_2}e^{-i\tilde{\omega}_{e_k g}\tau_3}G(\tau_3) \quad \text{(S4)}$$

In the present work we have considered Gaussian dephasing functions $G(\tau)$ with a standard deviation equal to 330 cm$^{-1}$, while the Lorentzian broadening is equal to 70 cm$^{-1}$.

The Raman and the probe pulses in the time domain can be expressed by anti-Fourier transforming

$$E_{R/P}(t) = \int_{-\infty}^{\infty} d\omega e^{-i\omega t} E_{R/P}(\omega) \quad \text{(S5)}$$

as a function of their representation in the frequency domain:

$$E_R(\omega) = E_R^{(0)}(\omega)e^{-i\omega_R \Delta T},\ E_P(\omega) = E_P^{(0)}(\omega)e^{i\sum_n C_n(\omega-\omega_P)^n} \quad \text{(S6)}$$

where $E_R^{(0)}(\omega)$ and $E_P^{(0)}(\omega)$ are the square of the RP-PP spectra, respectively. By Fourier transforming Eq. S4 over $t$, we can obtain the third order nonlinear polarization in the frequency domain, which reads as

$$P_A^{(3)}(\omega) = \left(\frac{i}{\hbar}\right)^3 \sum_{e_j e_k} \mu_{ge_j}\mu_{e_j g'}\mu_{g' e_k}\mu_{e_k g} \int_{-\infty}^\infty dt e^{i\omega t} \int_{-\infty}^\infty d\omega_3 \int_{-\infty}^\infty d\omega_2 \int_{-\infty}^\infty d\omega_1$$
$$E_P(\omega_3)E_R^*(\omega_2)E_R(\omega_1) \int_0^\infty d\tau_3 \int_0^\infty d\tau_2 \int_0^\infty d\tau_1$$
$$e^{-i\omega_1(t-\tau_1-\tau_2-\tau_3)}e^{i\omega_2(t-\tau_2-\tau_3)}e^{-i\omega_3(t-\tau_3)}e^{-i\tilde{\omega}_{e_j g}\tau_1}G(\tau_1)e^{-i\tilde{\omega}_{g' g}\tau_2}e^{-i\tilde{\omega}_{e_k g}\tau_3}G(\tau_3) \quad \text{(S7)}$$

Expressing also the inhomogeneous dephasing function in terms of their representation in

the frequency domain $G(\tau_1) = \int_{-\infty}^{\infty} d\omega_{D_1} e^{-i\omega_{D_1}\tau_1} G(\omega_{D_1})$, we obtain

$$P_A^{(3)}(\omega) = \left(\frac{i}{\hbar}\right)^3 \sum_{e_j e_k} \mu_{ge_j}\mu_{e_j g'}\mu_{g' e_k}\mu_{e_k g} \int_{-\infty}^{\infty} dt e^{i\omega t} \int_{-\infty}^{\infty} d\omega_3 \int_{-\infty}^{\infty} d\omega_2 \int_{-\infty}^{\infty} d\omega_1 \int_{-\infty}^{\infty} d\omega_{D_1} \int_{-\infty}^{\infty} d\omega_{D_3}$$
$$E_P(\omega_3)E_R^*(\omega_2)E_R(\omega_1)G(\omega_{D_1})G(\omega_{D_3}) \int_0^{\infty} d\tau_3 \int_0^{\infty} d\tau_2 \int_0^{\infty} d\tau_1$$
$$e^{-i\omega_1(t-\tau_1-\tau_2-\tau_3)} e^{i\omega_2(t-\tau_2-\tau_3)} e^{-i\omega_3(t-\tau_3)} e^{-i(\tilde{\omega}_{e_j g}+\omega_{D_1})\tau_1} e^{-i\tilde{\omega}_{g'g}\tau_2} e^{-i(\tilde{\omega}_{e_k g}+\omega_{D_3})\tau_3} \quad \text{(S8)}$$

By integrating over $t$, $\tau_1$, $\tau_2$ and $\tau_3$, we have

$$P_A^{(3)}(\omega) = \left(\frac{i}{\hbar}\right)^3 \sum_{e_j e_k} \mu_{ge_j}\mu_{e_j g'}\mu_{g' e_k}\mu_{e_k g} \int_{-\infty}^{\infty} d\omega_3 \int_{-\infty}^{\infty} d\omega_2 \int_{-\infty}^{\infty} d\omega_1 \int_{-\infty}^{\infty} d\omega_{D_1} \int_{-\infty}^{\infty} d\omega_{D_3}$$
$$G(\omega_{D_1})G(\omega_{D_3})E_P(\omega_3)E_R^*(\omega_2)E_R(\omega_1)\delta(\omega-\omega_1+\omega_2-\omega_3)$$
$$\frac{-1}{-i(\tilde{\omega}_{e_k g}+\omega_{D_3}-\omega_1+\omega_2-\omega_3)} \frac{-1}{-i(\tilde{\omega}_{g'g}-\omega_1+\omega_2)} \frac{-1}{-i(\tilde{\omega}_{e_j g}+\omega_{D_1}-\omega_1)} \quad \text{(S9)}$$

where the $\delta(\omega-\omega_1+\omega_2-\omega_3)$ represents the energy conservation and can be exploited to solve the integral over $\omega_3$:

$$P_A^{(3)}(\omega) = \left(\frac{1}{\hbar}\right)^3 \sum_{e_j e_k} \mu_{ge_j}\mu_{e_j g'}\mu_{g' e_k}\mu_{e_k g} \int_{-\infty}^{\infty} d\omega_2 \int_{-\infty}^{\infty} d\omega_1 \int_{-\infty}^{\infty} d\omega_{D_1} \int_{-\infty}^{\infty} d\omega_{D_3}$$
$$\frac{G(\omega_{D_1})G(\omega_{D_3})E_P(\omega-\omega_1+\omega_2)E_R^*(\omega_2)E_R(\omega_1)}{(\tilde{\omega}_{e_k g}+\omega_{D_3}-\omega)(\tilde{\omega}_{g'g}-\omega_1+\omega_2)(\tilde{\omega}_{e_j g}+\omega_{D_1}-\omega_1)} \quad \text{(S10)}$$

that can be expressed also as

$$P_A^{(3)}(\omega) = \left(\frac{1}{\hbar}\right)^3 \sum_{e_j e_k} \mu_{ge_j}\mu_{e_j g'}\mu_{g' e_k}\mu_{e_k g} \int_{-\infty}^{\infty} d\omega_1 \int_{-\infty}^{\infty} d\Delta \int_{-\infty}^{\infty} d\omega_{D_1} \int_{-\infty}^{\infty} d\omega_{D_3}$$
$$\frac{G(\omega_{D_1})G(\omega_{D_3})E_P(\omega-\Delta)E_R^*(\omega_1-\Delta)E_R(\omega_1)}{(\tilde{\omega}_{e_j g}+\omega_{D_1}-\omega_1)(\tilde{\omega}_{g'g}-\Delta)(\tilde{\omega}_{e_k g}+\omega_{D_3}-\omega)} \quad \text{(S11)}$$

introducing the new variable $\Delta = \omega_1 - \omega_2$.

By defining the RP preparation function

$$I_A(\Delta) = \sum_{e_j} \mu_{ge_j}\mu_{e_jg'} \int_{-\infty}^{+\infty} d\omega_1 \int_{-\infty}^{\infty} d\omega_{D_1} G(\omega_{D_1}) \frac{E_R(\omega_1)E_R^*(\omega_1-\Delta)}{(\omega_1-\tilde{\omega}_{e_jg}-\omega_{D_1})} \tag{S12}$$

$P_A^{(3)}(\omega)$ becomes

$$P_A^{(3)}(\omega) = \left(\frac{1}{\hbar}\right)^3 \sum_{e_k} \mu_{g'e_k}\mu_{e_kg} \int_{-\infty}^{\infty} d\omega_{D_3} G(\omega_{D_3})$$
$$\int_{-\infty}^{\infty} d\Delta \frac{E_P(\omega-\Delta)I_A(\Delta)}{(\tilde{\omega}_{g'g}-\Delta)(\tilde{\omega}_{e_kg}+\omega_{D_3}-\omega)} \tag{S13}$$

By similar steps, the nonlinear polarization of diagram $B_k$ can be expressed as

$$P_B^{(3)}(\omega,\Delta T) = \left(\frac{1}{\hbar}\right)^3 \sum_{e_k} \mu_{ge_k}\mu_{e_kg'} \int_{-\infty}^{+\infty} d\omega_{D_3} G(\omega_{D_3}) \tag{S14}$$
$$\int_{-\infty}^{+\infty} d\Delta \frac{I_{RB}(\Delta,\Delta T)E_P(\omega+\Delta)}{(\Delta+\tilde{\omega}_{gg'})(\tilde{\omega}_{e_kg'}+\omega_{D_3}-\omega)} \tag{S15}$$

with

$$I_{RB}(\Delta,\Delta T) = \sum_{e_k} \int_{-\infty}^{+\infty} d\omega_1 \frac{\mu_{e_kg}\mu_{g'e_k}E_R^*(\omega_1,\Delta T)E_R(\omega_1-\Delta,\Delta T)}{-\omega_1-\tilde{\omega}_{ge_k}} \tag{S16}$$

# Density Functional Theory calculations

Density functional theory (DFT) and TD-DFT calculations have been performed with CAM-B3LYP[7] functional and 6-311++G(2d,2p) basis set basis set with the Gaussian 09 software package. The normal modes eigenvectors calculated in the ground state have been exploited to generate seven displaced geometries along the different normal modes under consideration, which have then been exploited to calculate by TD-DFT the energy in the excited electronic state. In Fig. S4, we report the extracted PES along the different normal mode projections. The circles indicate the energies obtained from DFT and TD-DFT calculations, while the colored parabolas are parabolic fits. For the excited state continuous lines have

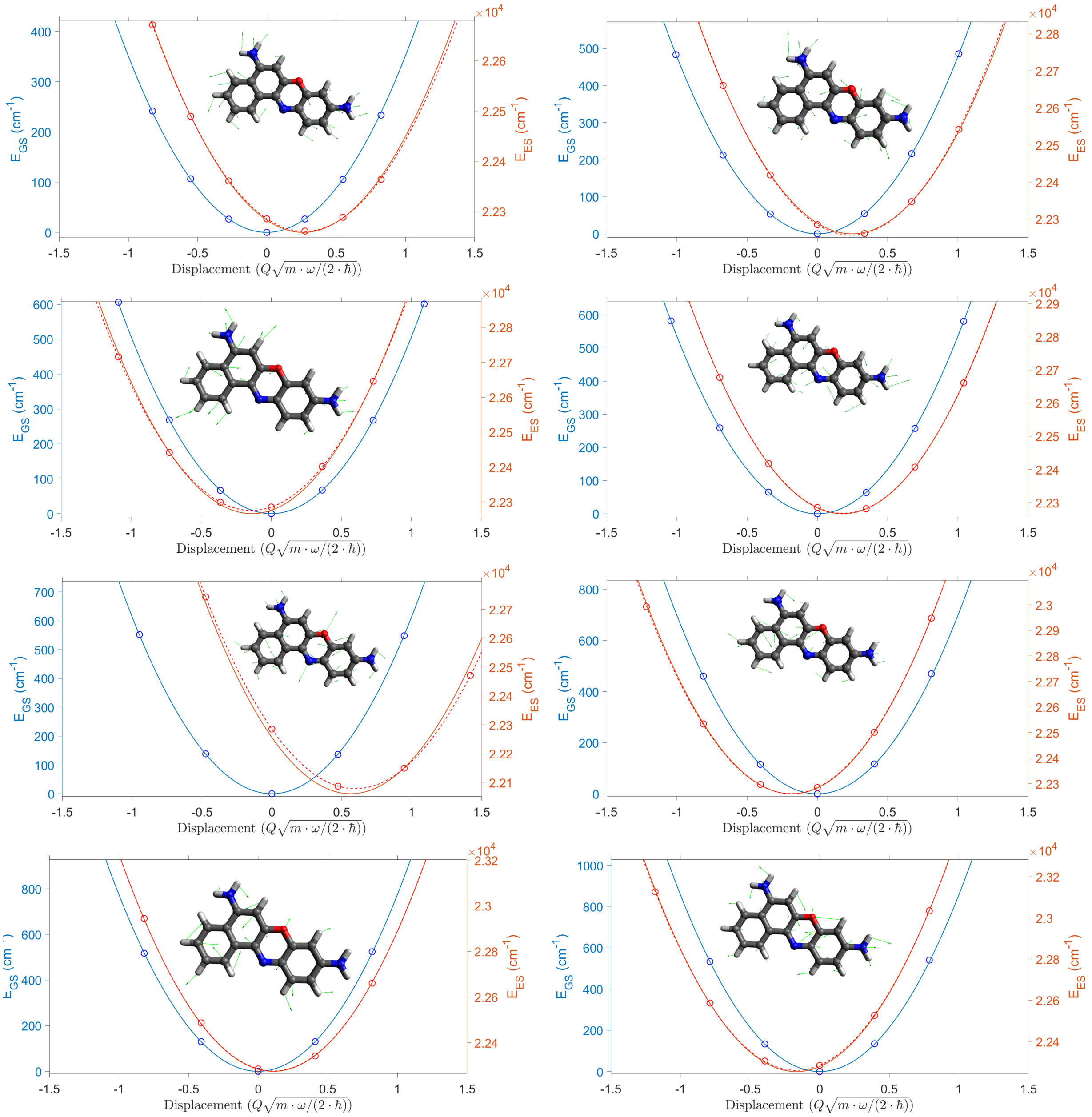


Figure S4: Potential energy surfaces, calculated using CAM-B3LYP functional and 6-311++G(2d,2p) basis set, are reported as a function of the nuclear displacement along different normal modes for the ground (blue lines) and excited (red) electronic states. The corresponding eigenvectors are reported as insets. The circles indicate the energies obtained from DFT and TD-DFT calculations, while the colored parabolas are a parabolic fit.

been extracted fixing the eigenfrequencies equal to the ground state, while for the dashed lines they have been set as a fitting parameter. In Fig. S5, the TD-DFT calculated electron density difference (EDD) map is reported in order to evaluate the relation between the electronic structure and excited state displacements. The EDD indicates a modification of the electron density in the excited state around the oxazine oxygen and nitrogen atoms, which have strong displacement along the 592 $cm^{-1}$ normal mode (Fig. S4).

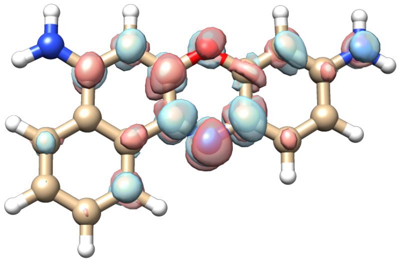

Figure S5: Electron density difference map between the ground and the excited state calculated using CAM-B3LYP functional and 6-311++G(2d,2p) basis set

**Table 1: Peak positions and displacements between ground and excited state PESs are reported with the corresponding 90% confidence intervals. In the third column we report the CAM-B3LYP eigenfrequencies obtained using the CAM-B3LYP functional and the 6-311++G(2d,2p) basis set,[7] while in the fourth one the corresponding displacements along the different normal coordinates.**

| $\tilde{\nu}_{g'g}$ ($cm^{-1}$) | $d = Q\sqrt{\frac{m\omega_0}{2\hbar}}$ | $\tilde{\nu}^{CAM-B3LYP}$ ($cm^{-1}$) | $d_{CAM-B3LYP}$ |
|---|---|---|---|
| 345 (2) | 0.17 (0.02) | 351 | 0.27 |
| 470 (2) | 0.20 (0.02) | 478 | 0.24 |
| 493 (2) | 0.13 (0.02) | 507 | 0.15 |
| 526 (2) | 0.26 (0.02) | 537 | 0.18 |
| 592 (2) | 0.64 (0.03) | 614 | 0.60 |
| 675 (2) | 0.19 (0.02) | 697 | 0.19 |
| 751 (2) | 0.17 (0.02) | 774 | 0.11 |
| 833 (2) | 0.17 (0.02) | 857 | 0.17 |

## References


(1) Leng, W.; Kelley, A. M. Resonance Raman Intensity Analysis of Cresyl Violet Bound to SiO2Colloidal Nanoparticles. *Langmuir* **2003**, *19*, 7049–7055.

(2) Mukamel, S. *Principles of Nonlinear Spectroscopy*; Oxford University Press: New York, 1995.

(3) Rahav, S.; Mukamel, S. Ultrafast Nonlinear Optical Signals Viewed from the Molecule's Perspective: Kramers-Heisenberg Transition-Amplitudes versus Susceptibilities. *Adv. At., Mol., Opt. Phys.* **2010**, *59*, 223–263.

(4) Dorfman, K. E.; Fingerhut, B. P.; Mukamel, S. Time-resolved broadband Raman spectroscopies: A unified six-wave-mixing representation. *J. Chem. Phys* **2013**, *139*, 124113.

(5) Batignani, G.; Fumero, G.; Mukamel, S.; Scopigno, T. Energy flow between spectral components in 2D broadband stimulated Raman spectroscopy. *Phys. Chem. Chem. Phys.* **2015**, *17*, 10454–10461.

(6) Batignani, G.; Ferrante, C.; Fumero, G.; Scopigno, T. Broadband impulsive stimulated Raman scattering based on a chirped detection. *J. Phys. Chem. Lett.* **2019**, 7789–7796.

(7) Yanai, T.; Tew, D. P.; Handy, N. C. A new hybrid exchange–correlation functional using the Coulomb-attenuating method (CAM-B3LYP). *Chem. Phys. Lett.* **2004**, *393*, 51–57.
